# OpenSTED: open-source dynamic intensity minimum system for stimulated emission depletion microscopy

**DOI:** 10.1117/1.NPh.11.3.034311

**Published:** 2024-06-12

**Authors:** Stephanie A. Pierce, Jordan Jacobelli, Katherine S. Given, Wendy B. Macklin, Juliet T. Gopinath, Mark E. Siemens, Diego Restrepo, Emily A. Gibson

**Affiliations:** a University of Colorado Anschutz Medical Campus, Department of Bioengineering, Aurora, Colorado, United States; b University of Colorado Anschutz Medical Campus, Department of Immunology and Microbiology, Aurora, Colorado, United States; c University of Colorado Anschutz Medical Campus, Barbara Davis Research Center, Aurora, Colorado, United States; d University of Colorado Anschutz Medical Campus, Department of Cell and Developmental Biology, Aurora, Colorado, United States; e University of Colorado Boulder, Department of Electrical, Computer, and Energy Engineering, Boulder, Colorado, United States; f University of Colorado Boulder, Department of Physics, Boulder, Colorado, United States; g University of Denver, Department of Physics and Astronomy, Denver, Colorado, United States

**Keywords:** stimulated emission depletion, dynamic minimum, super-resolution, fluorescence, microscopy

## Abstract

**Significance:**

Stimulated emission depletion (STED) is a powerful super-resolution microscopy technique that can be used for imaging live cells. However, the high STED laser powers can cause significant photobleaching and sample damage in sensitive biological samples. The dynamic intensity minimum (DyMIN) technique turns on the STED laser only in regions of the sample where there is fluorescence signal, thus saving significant sample photobleaching. The reduction in photobleaching allows higher resolution images to be obtained and longer time-lapse imaging of live samples. A stand-alone module to perform DyMIN is not available commercially.

**Aim:**

In this work, we developed an open-source design to implement three-step DyMIN on a STED microscope and demonstrated reduced photobleaching for timelapse imaging of beads, cells, and tissue.

**Approach:**

The DyMIN system uses a fast multiplexer circuit and inexpensive field-programmable gate array controlled by Labview software that operates as a stand-alone module for a STED microscope. All software and circuit diagrams are freely available.

**Results:**

We compared time-lapse images of bead samples using our custom DyMIN system to conventional STED and recorded a ∼46% higher signal when using DyMIN after a 50-image sequence. We further demonstrated the DyMIN system for time-lapse STED imaging of live cells and brain tissue slices.

**Conclusions:**

Our open-source DyMIN system is an inexpensive add-on to a conventional STED microscope that can reduce photobleaching. The system can significantly improve signal to noise for dynamic time-lapse STED imaging of live samples.

## 1 Introduction

Historically, the limit to the achievable spatial resolution of an optical microscope was defined by Abbe’s criteria based on principles of diffraction of light. With the development of high numerical aperture and low aberration optics, diffraction limited imaging modalities, including optical sectioning confocal fluorescence microscopy, have been used to investigate many interesting research questions in the biomedical field. However, many open questions in biology cannot be addressed without resolving finer details closer to the size of proteins and other biomolecules, typically tens of nanometers. Super-resolution (SR) microscopy techniques, such as stimulated emission depletion (STED),^1^^,^^2^ MINFLUX^3^^,^^4^ structured illumination microscopy (SIM),^5^^,^^6^ and single molecule localization techniques, such as PALM/STORM^7^^,^^8^ have moved fluorescence microscopy beyond the Abbe diffraction limit, allowing spatial resolutions of 10’s of nanometers. These SR microscopy techniques have opened up new possibilities for research using optical microscopy that were closed previously, examples of which include: observing the dynamics of dendritic spines in the brains of live zerbrafish larvae and mice,^9^ imaging the periodicity of the actin cytoskeleton using STORM in fixed neurons^10^ and STED in live neurons,^11^ and imaging the nanoscale distribution of Tom20 in mitochondria as a function of cell type, cell location, and colony location.^12^

STED microscopy, first demonstrated by the lab of Stefan Hell,^2^ has been utilized for live cell and *in vivo* SR imaging due to its fast times and ability to directly retrieve a super-resolved image without any required post-processing, as compared to PALM/STORM. STED microscopy can provide enhanced spatial resolution in comparison to SR-SIM. STED microscopy builds on conventional fluorescence microscopy by adding a second “STED” laser, tuned to the red tail of the fluorophore’s emission spectrum, co-aligned with the traditional excitation laser. The phase of the input STED beam is modified using a vortex waveplate to generate a donut spatial pattern at the focus of the objective, with a central null overlapped with the excitation focal spot. The co-aligned lasers are raster-scanned on the sample and the fluorescence is collected through a confocal pinhole to a photodetector. When a fluorophore is overlapped with the intense STED laser, outside of the central null, the stimulated emission rate is much higher than spontaneous emission, preventing fluorescence. The result is that only those molecules at the null in the STED donut beam can fluoresce, and thus be detected. With high STED laser intensities, resolution down to tens of nanometers is achievable in labeled biological samples.^13^^,^^14^

The resolution of a STED microscope is given as^15^

$$\Delta r=\frac{0.45\lambda }{\mathrm{NA}\sqrt{1+\frac{I}{I_{\mathrm{sat}}}}},$$

where NA is the numerical aperture of the objective, λ is the wavelength, I is the maximum intensity of the STED beam, and $I_{\text{sat}}$ is the saturation intensity of the fluorophore. To achieve as much as 10-fold improvement in resolution, the STED laser needs to be approximately three orders of magnitude higher power than the excitation laser (μW of excitation, mW of STED power). Unfortunately, high STED intensity can cause photobleaching, and this imposes a limit to the intensity and therefore the attainable resolution.^16^ For dynamic imaging, photobleaching also limits the amount of time that live biological processes can be monitored with STED. In conventional STED, the high-power STED laser is scanned continuously over the sample regardless of whether there are fluorophores at a given pixel in the image, leading to excess sample photobleaching.

In recent years, several methods have been developed to reduce photobleaching during STED imaging, including RescueSTED^17^ and dynamic intensity minimum (DyMIN).^18^ DyMIN, first demonstrated in 2017 by S. Hell, is an improvement on RescueSTED. DyMIN dynamically controls the STED laser intensity pixel by pixel to minimize the power on the sample. If there is no fluorescence detected from a pixel, the STED laser power remains off and the beam is moved to the next pixel. If fluorescence is detected above a threshold set for each DyMIN step, the DyMIN algorithm will incrementally increase the STED power. Ideally, the maximum STED power is only used when a fluorophore is present at the STED null, lower power is used when the fluorophore is located off of the null, and no STED power is used when no fluorophore is present. The DyMIN process can greatly reduce the overall exposure of the sample to the STED laser, especially for sparsely labeled samples, and results in decreased photobleaching. DyMIN can therefore allow acquisition of a time series over a longer period before the sample is photobleached, above a threshold set for each DyMIN step.

The implementation of DyMIN on a STED microscope can involve substantial effort, including programming an field-programmable gate array (FPGA) to implement the DyMIN algorithm and combining it with custom electronics to allow fast switching of the STED optical power by modulating the radio frequency (RF) power controlling an acousto-optic modulator (AOM). Here, we discuss our implementation using inexpensive components and provide schematics and source code to make DyMIN simpler to use for labs employing STED microscopy. Previous biological applications of the DyMIN method include imaging synaptic vesicles^19^ on fixed samples, in addition to a related adaptive illumination method that used an FPGA to rapidly turn off the STED laser when scanning over highly reflective parts of malaria cells, which are sensitive to light.^20^ However, the DyMIN code and detailed methods used in these papers were not open-source. An open-source DyMIN implementation will bring this powerful method to more researchers using STED and assist with other adaptive illumination schemes.

In the following sections, we present our custom DyMIN system and demonstrate its application to timelapse STED imaging, quantifying a reduction in photobleaching of beads. We also apply DyMIN to imaging of live cells and neural tissue samples.

## 2 Background: three-Step DyMIN Algorithm

**Fig. 1:**
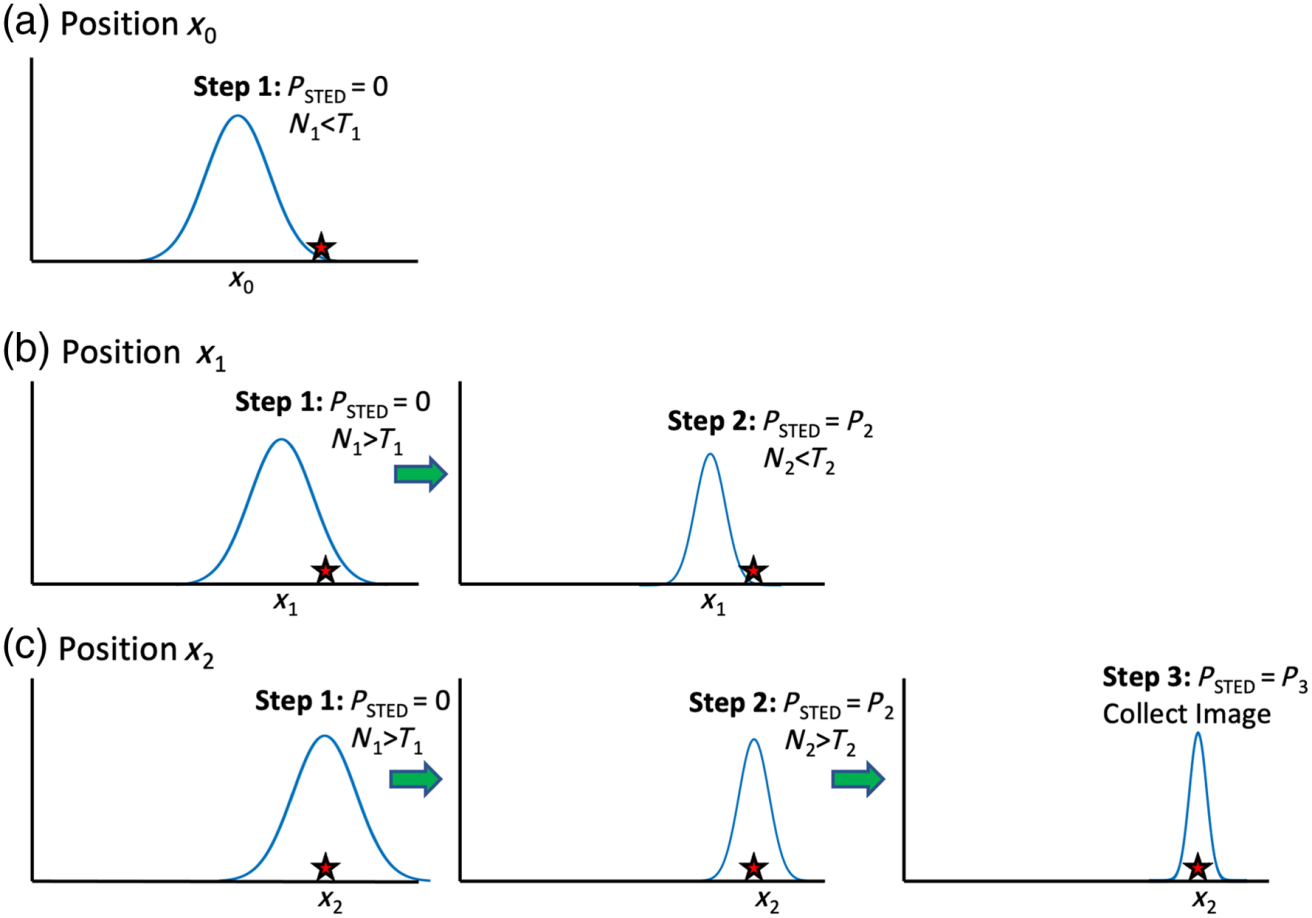
Three-step DyMIN STED algorithm process shown for three different positions as the scan approaches a fluorophore. (a) The initial step collects photons with no STED power applied at $x_{0}$. Because the fluorophore is outside the collection region, the photons counted ($N_{1}$) are less than the user supplied threshold for that step ($T_{1}$) so the process is aborted after step 1 and the scan moves to the next position, $x_{1}$. (b) The number of counts achieves threshold ($N_{1}>T_{1}$) for step 1 and step 2 initiates, where the STED power ($P_{2}$) is applied. However, with the smaller PSF the number of fluorescent photons collected does not meet the threshold ($N_{2}<T_{2}$), and the process is aborted after step 2. The beam is scanned to position $x_{2}$, shown in panel (c), where it overlaps with the fluorophore. In this case, the thresholds for step 1 and step 2 ($N_{1}>T_{1}$ and $N_{2}>T_{2}$) are met and the final DyMIN step, step 3, is implemented. $N_{3}$ photons are collected with the maximum STED power ($P_{3}$) and recorded at that position.

The DyMIN algorithm performs up to three steps of sequentially increasing STED laser power based on the number of collected fluorescent photons at each step, for each pixel in the image. Figure 1 illustrates the DyMIN algorithm process. In practice, the user selects a STED power value ($P_{n}$), fluorescence collection time ($t_{n}$), and a counts threshold ($T_{n}$), for each DyMIN step (n=1,2,3). The excitation power ($P_{\mathrm{EXC}}$) is held constant during imaging for our implementation of DyMIN.

At the nth DyMIN step, the STED power $P_{n}$ and excitation power $P_{\mathrm{EXC}}$ are incident on the sample, centered on a particular pixel, whereas the fluorescence photons are collected for a time $t_{n}$. The total number of photons collected is denoted $N_{n}$. Typically, the first DyMIN step is performed without any STED enhancement, i.e. $P_{1}=0\;\mathrm{mW}$, and successive DyMIN steps have increasing STED power, i.e., $P_{n\;}>P_{n-1}$. If at DyMIN step n, the photon counts fail to reach the predefined threshold ($N_{n}<T_{n}$), the algorithm is aborted, and the number of photon counts is set to zero for that pixel. Subsequently, the STED laser returns to zero power and the algorithm waits for the next pixel clock and then starts over at step n=1. If $N_{n}>T_{n}$ then the DyMIN algorithm stays on the pixel but moves on to the next DyMIN step, n+1, by increasing the STED power to $P_{n+1}$, collecting and counting the fluorescence photons $N_{n+1}$ and once again comparing the number of collected photons to the threshold value $T_{n+1}$. If the final DyMIN step (n=3) is reached, the highest STED laser power ($P_{3}$) illuminates the sample and the number of photons detected during the final step is recorded at that pixel.

Figure 1 outlines the three-step DyMIN algorithm for different scan positions approaching the location of a fluorophore. At the first location $x_{0}$ in Fig. 1(a) the fluorophore is outside the PSF of the excitation beam. The DyMIN algorithm starts at step 1, illuminates the sample with zero STED power, i.e., with only the confocal excitation, collects $N_{1}<T_{1}$ and thus stops after step 1, recording zero counts at that pixel location in the image. The DyMIN algorithm scans to next pixel, starting the DyMIN steps over with step 1 at location $x_{1}$. In Fig. 1(b), the scan is now centered at position $x_{1}$, and the fluorophore is within the confocal PSF, thus the number of photons collected exceeds the threshold $N_{1}>T_{1}$ and the DyMIN algorithm moves on to Step 2, changing the STED power to $P_{2}$. The algorithm collects photon counts for a duration $t_{2}$, but the effective PSF is smaller and there is no longer any overlap with the fluorophore position, thus the threshold is not reached ($N_{2}<T_{2}$), the DyMIN steps are aborted, and the number of photons counted is recorded as zero at that pixel. The STED laser is reset to zero power and the DyMIN algorithm proceeds to the next pixel to start over at step 1. In Fig. 1(c) the center of the beam fully overlaps with the fluorophore at position $x_{2}$, thus the counts are above threshold for steps 1 and 2, and step 3 is reached, the final DyMIN step. The number of photons collected at step 3, at the maximum STED power $P_{3}$, will be counted over a collection time of $t_{3}$ and recorded.

## 3 Methods

### 3.1 DyMIN STED Implementation

**Fig. 2:**
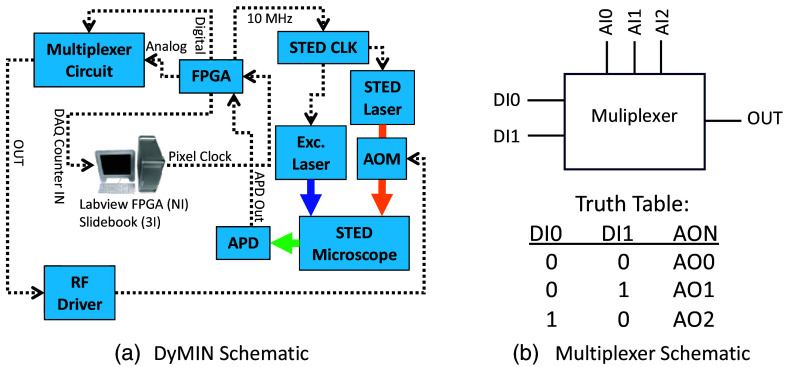
Hardware implementation of the DyMIN add on to our STED microscope. (a) An FPGA runs the DyMIN algorithm. A 10 MHz output from the FPGA is used to synchronize a function generator (STED CLK) that triggers the STED and excitation laser. The inputs to the FPGA are the digital photon counting signal from the avalanche photodiode (APD) and a pixel clock from the computer DAQ controlled by software for image acquisition. The FPGA then outputs the modified APD counter signal back to the computer DAQ as the photon signal recorded by the acquisition software. Additionally, three analog outputs and two digital outputs from the FPGA are sent to a multiplexer circuit to control the STED laser power at the different DyMIN steps. (b) A simplified schematic of the multiplexer and truth table. The circuit outputs the analog value (AI0-2) for the different DyMIN steps based on the digital inputs (DI0-1). The output from the multiplexer controls the AOM RF driver, to select the appropriate STED laser power.

Figure 2(a) shows a schematic for our implementation of the DyMIN on a STED microscope. The custom STED microscope is described in further detail in Sec. 3.1. An AOM controls the STED laser power (Newport-EOS part # N24080). An RF driver with an analog amplitude modulation input (Newport-EOS part # N21080-0.9AMVCO) controls the AOM, which allows fast switching of the STED laser power in ∼500  ns. The fluorescence signal is detected with an avalanche photodiode (APD, Excelitas SPCM-AQRH-15-FC) and the individual detected photons are converted to TTL pulses that are sent to the digital input on a field-programmable gate array (FPGA, NI myRIO-1900) programmed with the DyMIN algorithm using Labview FPGA (NI LabVIEW 2016, 32 bit). The FPGA counts the photons for each DyMIN step and then determines whether to go to the next step and raise the STED power or abort the DyMIN steps and move to the next pixel position.

The analog output on the FPGA has a maximum slew rate of 0.3  V/μs, too slow to directly control the AOM to change the output STED laser power on a time frame less than the dwell time used during imaging. Instead, we rely on the digital outputs from the FPGA to control a digital multiplexer circuit to rapidly modulate the control voltage to the AOM. A basic schematic of the multiplexer is shown in Fig. 2(b). Two digital outputs from the FPGA [labeled “Digital” in Fig. 2(a), DI0, DI1 in Fig. 2(b)] control the multiplexer (Analog Devices Inc, ADV3221 on ADV321-EVALZ) to switch between three different voltage values supplied by the analog outputs from the FPGA [labeled “Analog” in Fig. 2(a), AI0-AI2 in Fig. 2(b)]. Depending on the truth table in Fig. 2(b), the selected analog output [OUT in Figs. 2(a) and 2(b)] is sent from the multiplexer to the AOM RF driver, which allows faster control of the STED laser power than would be achievable using the analog output of the FPGA directly. The analog output values from the FPGA are chosen in software to select the STED power for the different DyMIN steps, $P_{n}$, determined by calibration.

In our implementation, we use Slidebook (Intelligent Imaging Innovations, Inc.) software for image acquisition and to control the scanning mirrors, however our DyMIN circuit and FPGA can be implemented on any image acquisition software that has a pixel clock and photon counting input. On our system, the pixel clock is supplied by a data acquisition (DAQ) board (NI PCIe-6259) to the FPGA digital input channel to synchronize the DyMIN steps with the image acquisition. The modified/unmodified (DyMIN/conventional STED) photon counts at each pixel are sent by a digital TTL output from the FPGA to the counter input on the DAQ to record photon counts in the software. For DyMIN, only the photon counts for the final DyMIN step are sent to the DAQ. Finally, we use a 10 MHz output clock signal from the FPGA to synchronize the STED laser clock so that the number of laser pulses per step stays constant over the measurement.

The FPGA code and further details on the electronics are publicly available for download.^21^

### 3.2 Details of the DyMIN STED Microscope

**Fig. 3:**
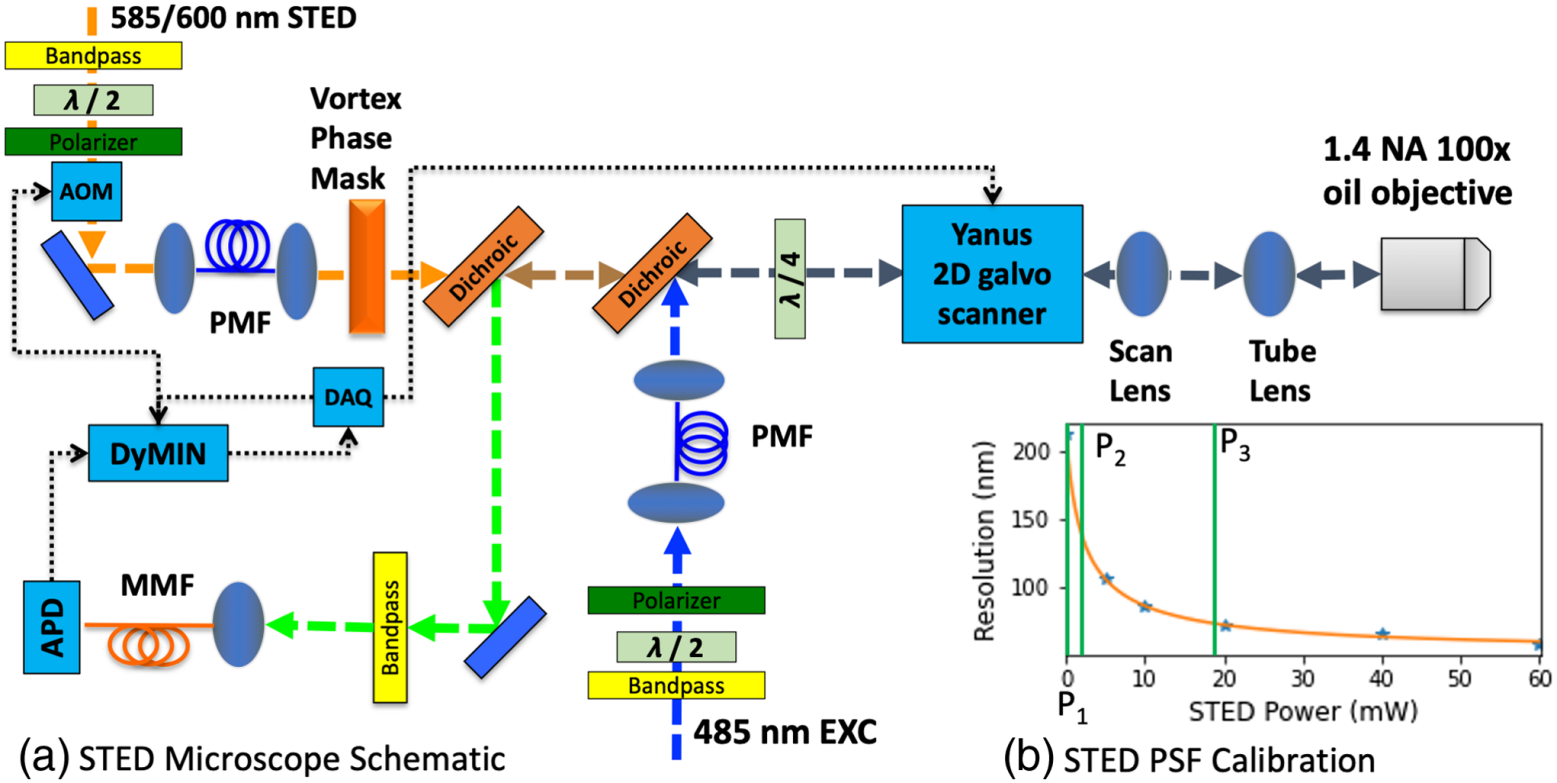
(a) Schematic of custom-built STED microscope for imaging YFP/GFP with 485 nm excitation laser and 585/600 nm STED laser. Both excitation/STED beams pass through a bandpass filter and a half waveplate and polarizer for power control. An AOM in the STED laser path controls the STED power for the DyMIN algorithm. The lasers are each coupled into a PM fiber, collimated, and the STED laser is sent through a vortex phase mask before being combined with the excitation laser using a dichroic. A quarter waveplate sets the polarization to be circular, followed by the Yanus 2D galvo scanning system, scan lens, tube lens, and objective lens, which focuses the beams onto the sample. The resulting fluorescence is selected using a dichroic and bandpass filter, coupled into a multimode fiber that acts as a confocal pinhole, then sent to the APD. (b) The STED resolution calibration curve. The resolution of the STED microscope at a series of STED laser power values was estimated by imaging 23 nm fluorescent beads. These values were fit to a curve (orange line) using Eq. (1). Example values from our bead imaging experiments of $P_{1}$, $P_{2}$, and $P_{3}$ are indicated with green vertical lines.

Our custom-built beam-scanning STED microscope for imaging yellow fluorescent protein (YFP) and green fluorescent protein (GFP) in live cells is shown schematically in Fig. 3. The excitation laser is a 485 nm pulsed diode laser (Picoquant, LDH-P-C-485-B controlled by PDL 800-B) and a 585 nm or 600 nm selectable pulsed output of a Rainbow laser (Mobius Photonics) serves as the STED laser. Both lasers are triggered by the same external pulse generator. The pulse widths of the STED and excitation laser are 800 ps and less than 140 ps, respectively. Both excitation and STED lasers pass through bandpass filters to clean up the spectra (Chroma, ET470/30X and ET600/40M) and a half-waveplate and a polarizer are used for power control. Each laser is then coupled into a PM fiber to clean up the spatial profiles. A vortex phase mask (RPC Photonics, VPP-1a) applies a spiral phase pattern to the STED laser to generate a donut shaped intensity pattern at the focal plane of the microscope. One dichroic beamsplitter (Chroma, ZT488RDC) combines the excitation and STED lasers while another (Chroma, ZT568RDC-XR) separates the fluorescence emission. A quarter waveplate (B. Halle, RAC 3.4.15), carefully aligned to obtain nearly perfect circular polarization of the STED beam (see Ref. 21 for details), is placed in the combined beam path of the excitation and STED beam before the galvo-scanner (Till Photonics, Yanus IV). The galvo-scanner is designed to image the first mirror onto the second mirror so that the STED beam remains stationary at the back focal plane of the objective during scanning.

A scan lens (Thorlabs, LSM03-VIS) and tube lens (internal to Olympus IX-71) image the second galvo mirror onto the back focal plane of the 100X/1.4NA oil immersion objective lens (Olympus, uPlanSApo) that focuses the beams on the sample. The objective is mounted on the turret of an Olympus IX-71 microscope platform, which has been modified to incorporate a 3 mm thickness protected silver mirror that sends light that comes into the left side port straight up through the center of the tube lens and objective lens. A Nano-ZL XY microstage (Mad City Labs) controls the position of the sample and widefield epifluorescence, brightfield, or DIC imaging through the oculars is used for alignment. A Nano-Z100 piezo nanopositioning system (Mad City Labs) provides axial translation to perform z-stacks or x-z and y-z scans required for the STED alignment process, as detailed in Ref. 22, with insert adapters for different sample holders. The fluorescence collected by the objective is de-scanned and reflected by the dichroic beamsplitter to a lens that focuses the fluorescence into a multimode fiber that serves as a confocal pinhole (Thorlabs, AC254-200A and M43L01). The fiber relays the fluorescence to the APD (Excelitas, SPCM-AQRH-15-FC), which sends output TTL pulses to the FPGA running the DyMIN code and subsequently to the DAQ on the computer for photon counting, as described above.

For DyMIN operation, the FPGA and multiplexer send an analog voltage signal to an AOM in the STED laser path to modulate the intensity. After the AOM, the STED laser is focused into a PM fiber. In characterizing the power control of the DyMIN and AOM, we found that the power level of the STED beam measured after the PM fiber has a time-dependent response after the AOM was switched on (see Fig. S1 in the Supplementary Material). The power stays constant over ∼10  ms, then a gradual drop is seen, followed by an increase to eventually reach a constant average power by ∼100  s. We believe that this slower power modulation is due to heating that influences the coupling efficiency into the fiber, likely due to the laser mode not being perfectly ${\mathrm{TEM}}_{00}$.

Therefore, less effective power is used for DyMIN compared with conventional STED if the same analog voltages are sent from the FPGA to the multiplexer and RF driver. To make comparisons between STED and DyMIN imaging, we carefully measured the steady state power (as would be used for STED when the laser is constantly on) and the power in the first 10 ms after the AOM is switched on. To compare photobleaching rates with our bead samples, we made sure to use a lower power for the STED beam when doing conventional STED so that we had the same effective power on the sample during the last DyMIN step.

### 3.3 Selecting the DyMIN Parameters

DyMIN has clear benefits for dynamic time-series STED imaging due to decreased photobleaching. To maximize resolution and minimize exposure to STED light during scanning, the DyMIN three-step parameters $P_{2}$, $t_{1}$, $t_{2}$, $T_{1}$, and $T_{2}$ need to be optimized. For the remainder of this paper, we will write these parameters as threshold photon counts $T=(T_{1},T_{2})$, fluorescence collection times $t=(t_{1},t_{2},t_{d})$ where $t_{d}$ is the time used for the final DyMIN step and also the effective dwell time for the recorded image, and power levels $P=(P_{1},P_{2},P_{3})$ where $P_{1}=0$ and $P_{3}$ is the final STED power. Below we outline how to set the intermediate parameters to achieve the desired final resolution while minimizing photobleaching during the DyMIN steps. The excitation power and STED power for the final DyMIN step are the same as what is used in conventional STED microscopy, with a pixel size of at least half the resolution required to achieve Nyquist frequency sampling.

#### 3.3.1 Power parameters

To determine the DyMIN step power levels, first the resolution versus STED power was calibrated by imaging 23 nm fluorescent beads at a series of STED laser powers. Individual beads were identified from raster-scanned images, and a 2D Lorentzian function was then fit to this data using the method of least squares. The STED resolution was then determined by taking the mean FWHM values for all beads in the image. The values were then fit to a curve using Eq. (1) and plotted in Fig. 3(b).

To determine the power levels for three-step DyMIN imaging of fluorescent beads, we first pick the resolution and subsequently the STED power to use for imaging, and this is the value for $P_{3}$. To determine the value for $P_{2}$, ideally one would like to minimize the STED light dose on the sample. In the original DyMIN manuscript (Ref. 18), the authors experimentally found the number of frames they could acquire before the signal dropped by 75% in a bead sample as a function of $P_{2}$. The value of $P_{2}$ that maximized the number of frames was then used. Note that the value of $P_{2}$ also depends on the final STED power used. Based on our final STED power of 18.7 mW, we tested two $P_{2}$ values (3 mW, 8 mW); however, we did not see a significant difference in photobleaching rate [see Figs. 5(a) and 5(b)].

#### 3.3.2 Collection times

For the step 1 and step 2 collection times ($t_{1}$ and $t_{2}$), we used Eq. (S1) in Ref. 18. The dwell time for the final step ($t_{d}$) is a tradeoff between photobleaching and signal to noise ratio (SNR). If the goal is to take a long image sequence, you want to minimize the dwell time to avoid photobleaching, however this will also reduce SNR. For the bead samples, we used a final dwell time of 10  μs and for the live cell and tissue we used 10 and 4  μs, respectively.

#### 3.3.3 Threshold parameters

To aid in the selection of the count thresholds for steps 1 and 2, we took a confocal image and a STED image at $P_{2}$ with the calculated collection times. We calculated 1/e of the peak fluorescence value of those images for use as $T_{1}$ and $T_{2}$, respectively, as suggested in Ref. 17. However, we found that for time-series imaging we needed smaller threshold values to reach threshold on the later images in a series, after the detected signal has dropped with photobleaching. This step would not be required for taking a single DyMIN image. An improvement would be to allow the threshold to decrease dynamically over time during a time series based on the fluorescence in the most recent image, but we did not explore that option for this paper.

### 3.4 Sample Preparation

For the DyMIN bead images, 45 nm Yellow-Green FluoSpheres™ (ThermoFisher Scientific, F8795) were diluted by 1:5000 in 1% phosphate buffered saline (PBS) sonicated for 10 min, dispersed on a poly-l-lysine coated coverslip for 5 min, washed and then immersed in Prolong Glass (ThermoFisher Scientific, P36982) immersion media and attached to a microscope slide. A commercially prepared sample slide with 23 nm DNA origami beads labeled with Atto 488 was used for STED resolution calibration (Gattaquant GmbH, GATTA-Beads B – brightline).

For the live T cell samples, lymph nodes and spleen were harvested from LifeAct-GFP donor mice. The lymph nodes and spleen were mechanically dissociated to a single cell suspension. T cells in the suspension were then activated by plating on anti-CD3 and anti-CD28 antibody (2  μg/mL each) coated tissue culture plates. Two days after activation, the T cells were transferred to culture flasks and further grown in complete Roswell Park Memorial Institute (RPMI) media containing 10  U/mL of IL-2 for 3 more days. On day 5 post-activation, the T cells were used for microscopy studies. For the imaging, the activated T cells were plated on chambered coverslips (μ-Slide 8 well from IBIDI) coated with 10  μg/mL of recombinant ICAM-1 (R&D systems) in RPMI media without Phenol Red and with 2% (wt/vol) BSA and 10mM HEPES. The T cells were then imaged at 37°C after allowing them to adhere to the ICAM-1 coated surface for at least 1 h.

The live tissue that was imaged was an organotypic cerebellar explant culture. To prepare the samples ex vivo cerebellar explants were prepared as previously described.^23^ Cerebella were dissected from P10 PLP-EGFP mice^24^ (Jax#33357), sliced at 300 mm on a McIlwain Tissue Chopper, and cultured on MilliCell 0.4  μm membrane inserts (Millipore) in media containing 15% Hank’s balanced salt solution, 15% heat-inactivated horse serum, 68% minimum essential media, 125 mM HEPES, 28 mM D-Glucose, 2 mM L-Glutamine, and 10,000  U/ml penicillin/streptomycin (Life Technologies) at 37°C. Media were exchanged every 2-3 days. Slices were incubated for 10 days prior to imaging. For imaging, slices were cut out of their inserts and placed tissue side down into a 35 mm glass-bottom dish.

For the live cell and tissue measurements, we used a Tokai HIT heated stage to keep the cells and tissue at 37°C during the measurements. All experiments were performed according to protocols approved by the University of Colorado Anschutz Medical Campus Institutional Animal Care and Use Committee.

## 4 Results: DyMIN Imaging of Fluorescent Beads and Live Cells and Tissue

To demonstrate our DyMIN implementation, we imaged 45 nm fluorescent bead samples. Five different 50-image sequence were taken with conventional STED and DyMIN STED on different areas of the sample with a field of view (FOV) of 10  μm×10  μm at 512  pixels×512  pixels, with 15 s between the start of each image. We took DyMIN sequences at four different combinations of the parameters, labeled DyMIN1-DyMIN4; see Table 1 for the parameters of each sequence. Note that all DyMIN sequences had the same step times and final step effective power, and the dwell time and STED power for the conventional STED sequence was the same as the final DyMIN step time and effective power. A larger FOV confocal image centered on the same field as the sequence (FOV=30  μm×30  μm, 512  pixels×512  pixels) was acquired before and after each sequence so that the bleaching before and after the image sequence could be measured.

**Table 1:** Parameters used for DyMIN and STED sequences.

Sequence	Step/dwell time(s) (μW)	Effective STED power(s) (mW)	Thresholds (counts)	Confocal intensity after/before
DyMIN1	(3.6625, 3.825, 10)	(0, 2.3, 18.7)	(4, 4)	0.446
DyMIN2	(3.6625, 3.825, 10)	(0, 2.3, 18.7)	(5, 5)	0.387
DyMIN3	(3.6625, 3.825, 10)	(0, 8.8, 18.7)	(4, 4)	0.454
DyMIN4	(3.6625, 3.825, 10)	(0, 8.8, 18.7)	(5, 5)	0.357
STED	10	18.7	NA	0.310

**Fig. 4:**
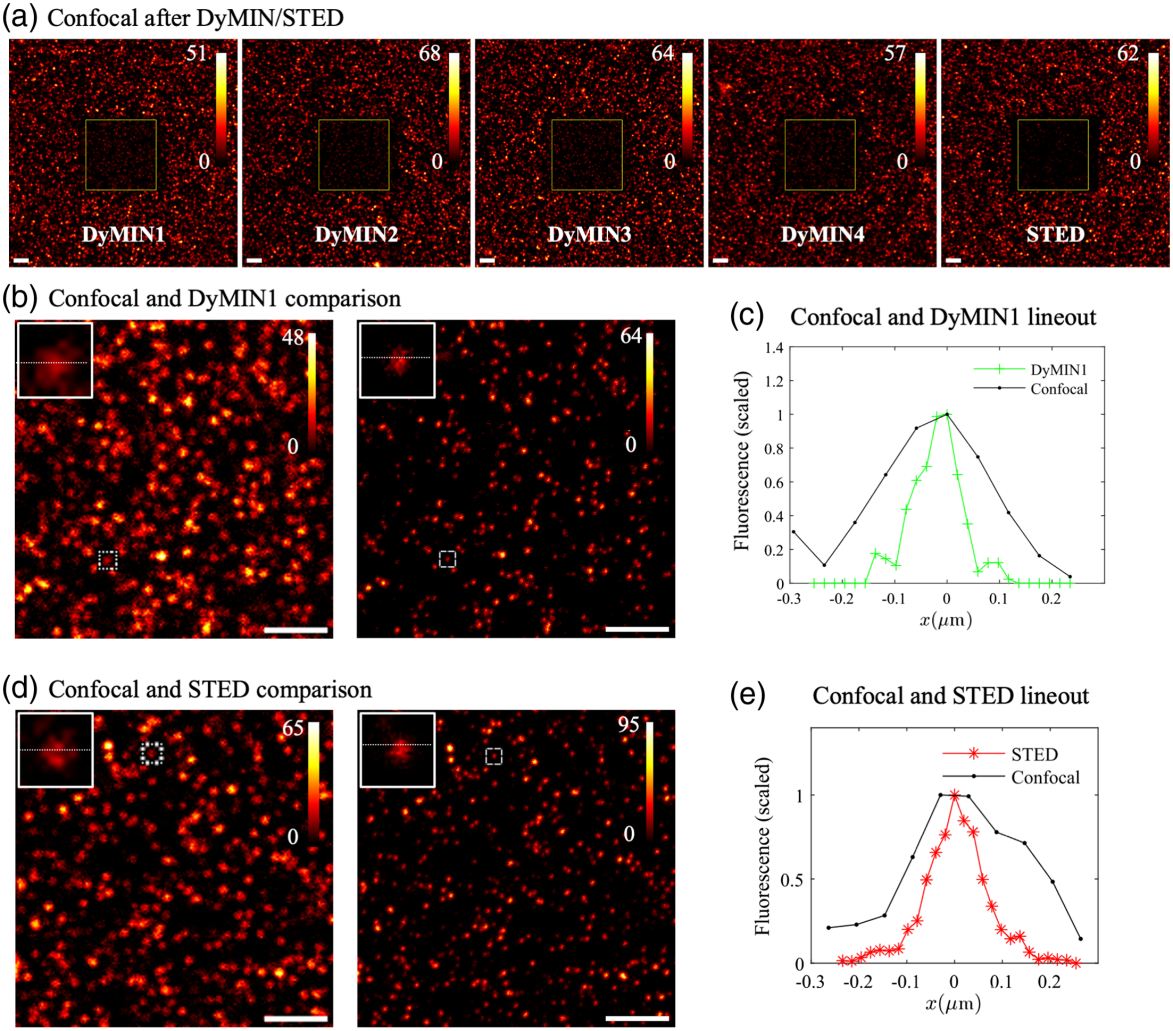
(a) Confocal images of a 30×30  μm FOV centered on the same location where DyMIN1-DyMIN4 and conventional STED 50-image sequences (see Table 1 for imaging parameters) were taken of a 45 nm fluorescent bead sample with 10×10  μm field and 512×512  pixels, after the DyMIN/STED sequences were acquired, as labeled. (b) Confocal image versus DyMIN1 image with a select bead lineout shown in panel (c) indicating a smaller FWHM of the bead with DyMIN super resolution imaging. Similar results are found for DyMIN2-4 (not shown). (d) Confocal image versus conventional STED image with a selected bead lineout shown in panel (e) with similar reduction in the FWHM. Colorbars are scaled to the maximum value of the image. Scalebars are 2  μm.

**Fig. 5:**
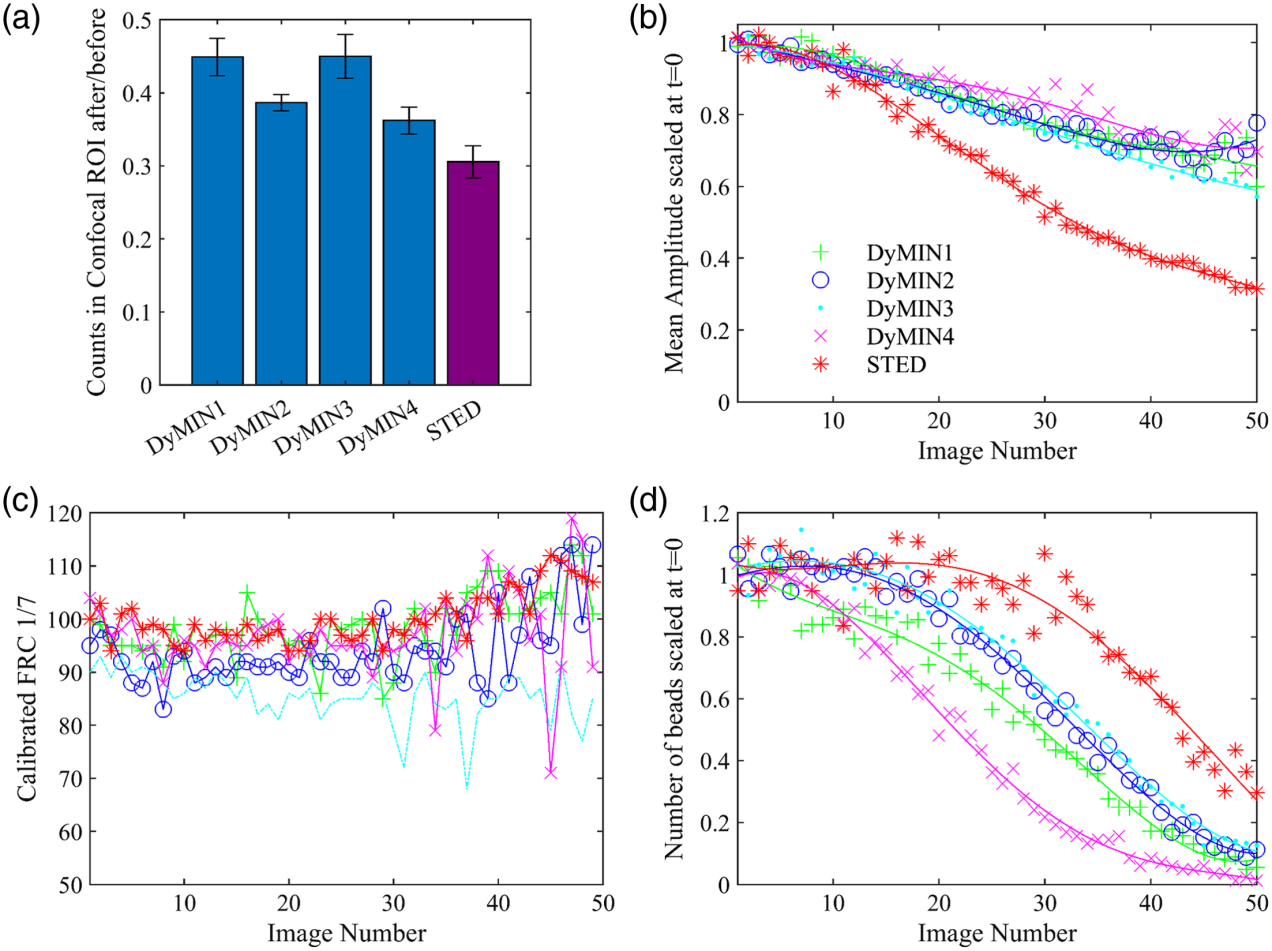
Analysis of photobleaching and resolution for DyMIN and STED images of fluorescent beads. (a) The ratio of the total confocal fluorescence counts in the DyMIN/STED imaging ROI [shown in Fig. 4(a)] after the 50-image sequence compared to before. Error bars were calculated by splitting the ROI into nine equal regions and calculating the standard deviation. (b) The mean amplitude of 2D Lorentzian fits of the beads verses image number, scaled to the mean of the initial three values. Fits were performed on a 15×15  pixel ROI around the bead center, selected using a peak finding routine in MATLAB. The lines are fourth degree polynomial fits to guide the eye. (c) The FRC, calculated with a fixed 1/7 threshold, verses image number, with the FRC calculated between subsequent images of the sequence (i,i+1), with individual curves for each image sequence. The close initial values indicate that the STED powers were equivalent during imaging. (d) The number of beads fit in panel (b) verses image number, scaled to the mean of the initial three values, also with a fourth degree polynomial fit. All traces in panels (b)–(d) use the color and marker scheme shown in the legend in panel (b).

Figure 4(a) shows the confocal image taken after each sequence with a larger FOV so the relative intensity compared to the unimaged regions can be seen. The fields that were imaged are dimmer than the surrounding field, but the photobleaching for all DyMIN sequences is lower than the STED comparison image. Figure 5(a) shows a bar graph of the ratio of the total confocal image counts in the regions of interest (ROIs) shown in Fig. 4(a) after to before the image sequence. The error bars indicate the standard deviation for the ratio, calculated by splitting the ROI into nine equal regions. The ratio for STED is lower than the ratio for all DyMIN sequences, with DyMIN maintaining 14% to 46% more of the initial fluorescence intensity, depending on the parameters selected. Note that the effective STED powers used in all these sequences are much lower than typically used in STED imaging, due to the lower effective STED power achieved during DyMIN (see Fig. S1 in the Supplementary Material), so these results are a lower bound on the bleaching prevented with DyMIN at the 2-3x higher STED powers typically used for imaging.

To characterize the DyMIN and conventional STED image sequence resolutions we calculated the fixed 1/7 threshold Fourier ring correlation (FRC)^25^^,^^26^ between subsequent images (i,i+1) in the sequence and plotted the results for each sequence on the same axes. The FRC was calculated using the ImageJ plugin available from the BioImaging and Optics Platform (PTBIOP) update site. Figure 5(c) shows the resulting FRC curves, with the curves starting near the same value, indicating we had equivalent STED powers during imaging for the sequences. Over the course of the sequences the fluorescence signal drops due to photobleaching and thresholding during previous steps, which causes larger variation in the FRC calculated at higher image numbers.

We performed Lorentzian fits on a 15-pixel ROI around each bead in the DyMIN/STED images and calculated the mean values of the amplitude of the beads over the course of the imaging sequence, scaled to the mean of the first three points. Figure 5(b) shows these plots, with the STED data showing a much faster decrease than the DyMIN traces. However, as shown in Fig. 5(d), the number of beads found by the fitting routine for the DyMIN traces decreases faster for all DyMIN sequences than for conventional STED. The DyMIN algorithm defines a pixel value at zero when the threshold is not reached. As a result, with changing sample intensities due to photobleaching, beads that are not bright enough to achieve threshold in steps 1 or 2 in DyMIN will disappear over time.

The DyMIN algorithm defines a pixel value at zero when the threshold is not reached. As a result, with changing sample intensities due to photobleaching, the effective size of the bead decreases since the threshold levels remain the same over time. Figure S7 in the supplement from Ref. 18 showed a similar effect: that DyMIN can lead to scan-dependent bleaching effects in the sample.

We next wanted to demonstrate that our DyMIN system could image live cells and will extend the number of images that can be acquired with good SNR. We imaged primary mouse T cells expressing lifeactGFP (a reporter for F-actin). In this sample, the T cells are very mobile and were relatively dim, making it a challenging sample for STED microscopy. We imaged a 23×23  μm FOV with 600×600  pixels, with 30 s between images at DyMIN effective powers P=(0,1.4,12.7)  mW. The DyMIN step time parameters used were t=(3.6,6.2,10)  μs. The 10  μs final dwell time was selected based on previous conventional STED time-lapse imaging on the samples, where we found it to be a reasonable balance between imaging speed and SNR. The thresholds selected were T=(3,4) counts.

**Fig. 6:**
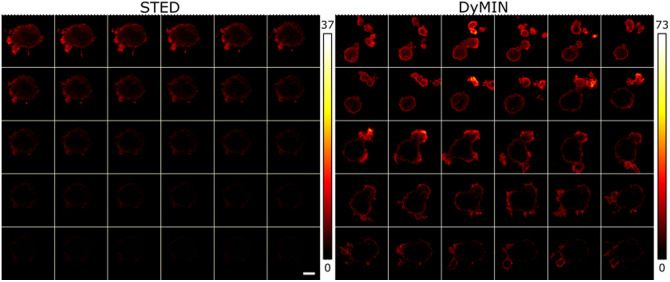
A sequence of 30 images (left to right, top to bottom), taken 30 s apart, with a 23×23  μm FOV with 600×600  pixels of wild type T cells expressing lifeactGFP. A 5  μm scalebar is shown in the bottom right STED image. The STED sequence on the left quickly bleaches so that by the middle of the sequence the image is no longer visible on the same scale as the first image, whereas the DyMIN sequence is still visible even on the 30’th image. The DyMIN settings were P=(0,1.4,12.7)  mW, T=(3,4) counts and t=(3.6,6.2,10)  μs. A dwell time of 10  μs was used for the conventional STED sequence at P=24  mW.

Although these low thresholds make it more likely for a pixel without significant fluorescence to be exposed to STED light in the first images of the series, it did allow a series of 30 images to be taken for DyMIN while maintaining good visibility of the sample (Fig. 6 right), whereas the conventional STED image series quickly bleaches so that the sample is no longer visible on the same intensity scale (Fig. 6 left). The conventional STED imaging was done using the same dwell time, 10  μs at a STED power of 24.0 mW.

**Fig. 7:**
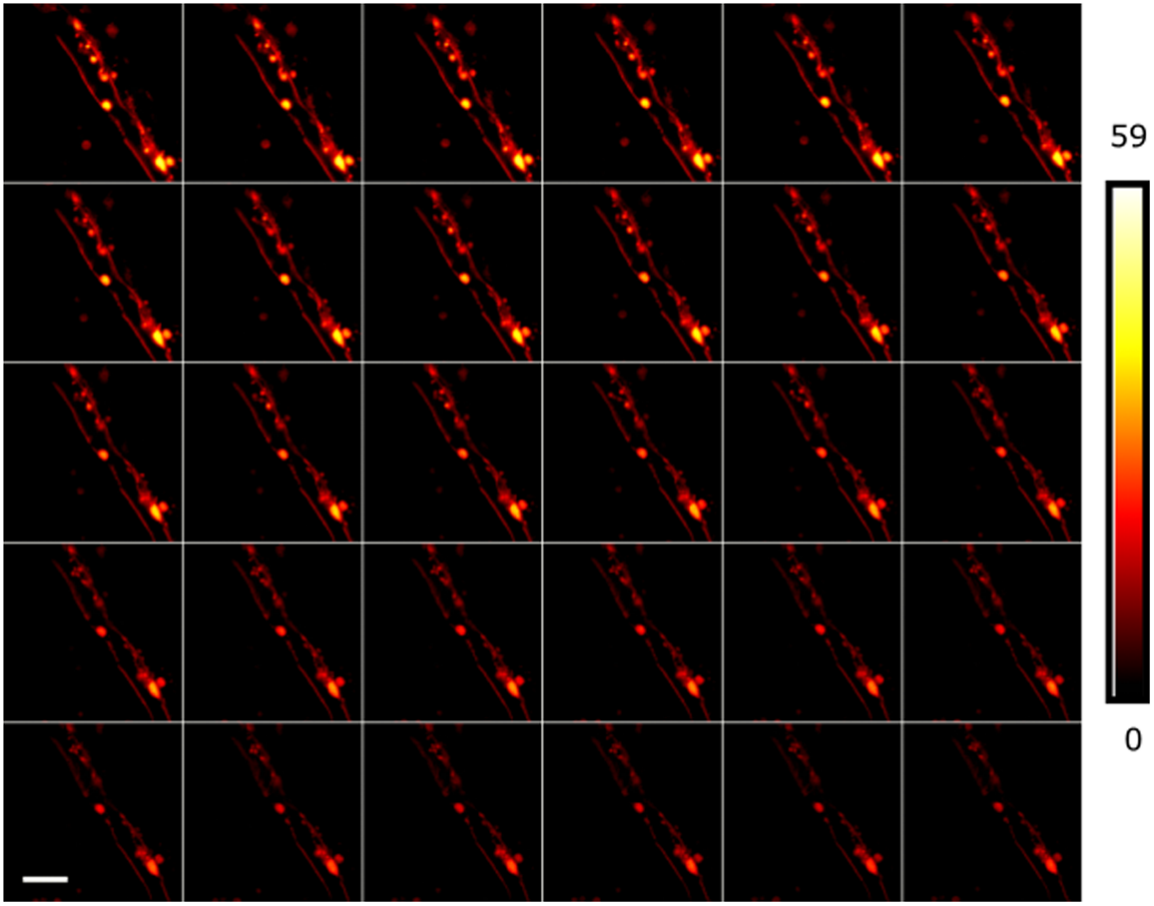
Time series images of a live cerebellar brain slice from a PLP-eGFP mouse using DyMIN imaging (left to right, top to bottom). A 20×23  μm FOV with 512×512  pixels was imaged using DyMIN parameters T=(12,12) counts, t=(3.6,5,4)  μs and with powers of P=(0,5,18.3)  mW. A 30 image timeseries was taken at 30 s between the start of each image. The sample remains visible throughout the entire imaging sequence. A 5  μm scalebar is shown in the bottom left image.

The final sample imaged was a cerebellar slice from a PLP-eGFP mouse prepared as described above. We imaged the tissue on a heated stage to maintain sample at a 37°C culturing environment. The parameters for DyMIN were T=(12,12) counts, t=(3.6,5,4)  μs and effective STED powers of P=(0,3.1,18.3)  mW, using a 20×20  μm FOV with 512×512  pixels. Figure 7 shows a timelapse imaging series of myelin along the length of an axon with 30 s total delay between the start of each image. The maximum fluorescence collected during the first image was 59 photons and that is the scale used to show the entire image series so that the effects of photobleaching will be evident. Although some bleaching is seen between the first and final images, the resulting series allows the sample to be imaged throughout the time series.

## 5 Discussion and Conclusions

We have demonstrated an open-source implementation of the DyMIN method first reported by the Hell group in 2017. The DyMIN add-on is an inexpensive FPGA programmed with LabVIEW FPGA and custom electronics. Open source DyMIN should allow implementation on any STED microscope with the only requirements being photon counting detection, which is generally used for STED imaging, a pixel clock signal from the image acquisition software to provide a start signal for the DyMIN algorithm at each pixel, and the space to add an AOM into the STED beam path.

DyMIN can be used to achieve increased resolution or signal-to-noise in 2D STED but is especially powerful for 3D STED imaging,^18^ or the focus here, time series imaging of live samples. We are interested in using DyMIN to extend the number of images we can take over the same field while imaging live cells and tissue, to observe dynamics as live samples interact with their environment or experience a photo-activation or uncaging laser that initiates a dynamical cellular process.

One disadvantage of using DyMIN in image sequences is that, to extend the image sequence, one must start with a smaller threshold than ideal so that later images still reach the threshold. Unfortunately, this choice causes more photobleaching during the first images of a sequence. One modification that would improve DyMIN for longer term image sequences would be to use the photon counts in an image to modify the DyMIN step thresholds dynamically during image sequences so that the threshold decreases over time.

Dynamic STED imaging of living samples interacting with the environment will be difficult to achieve without the ability to prevent significant photobleaching that DyMIN provides. The described DyMIN system will allow more research groups to implement this powerful technique in their labs and, by providing this in an open-source format, can help foster a community of active participation in microscopy technology development.

## Supplementary Material



## Data Availability

The code, custom electronics design, and the data in this article are available in a GitHub repository, with a link provided in Ref. 21.
