# Supplementary material for: OpenSTED: open-source dynamic intensity minimum system for stimulated emission depletion microscopy
Source: Neurophotonics. 2024 Jun 12;11(3):034311. doi: 10.1117/1.NPh.11.3.034311 (PMC11167952; doi:10.1117/1.NPh.11.3.034311)
Supplement: Supplementary file 1 [file NPh_011_034311_SD001.pdf]

OpenSTED: Open-Source DyMIN system for STED Microscopy

Supplementary Material

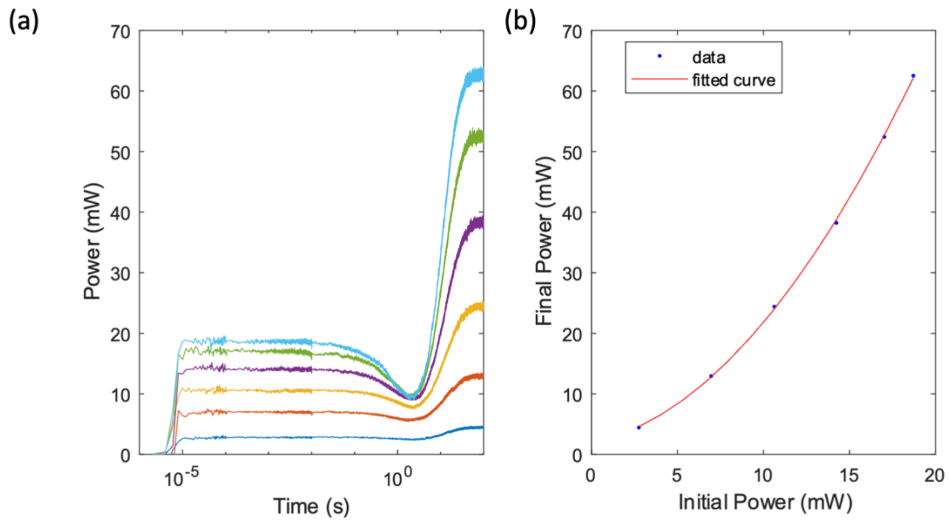

Figure S1. Measured power transients of STED laser. (a) STED laser power versus time on a logarithmic scale, measured after the PM fiber, when switching on the AOM from 0 to different power values. There is an initial steady response for ~10 ms followed by a decay and then a slow increase, likely due to heating of the input fiber tip causing changes to the coupling efficiency. The time-dependent response becomes more dramatic at higher power values. (b) The final power (steady state at long time scales) versus the initial power (within ~10 ms). The effective power during DyMIN is the initial power, after the AOM is switched on, while the effective power during conventional STED is the final power, where the laser power stays on continuously during imaging. A quadratic fit of the final power values verses initial power is shown in red.
